# Imidazopyridine Compounds Inhibit Mycobacterial Growth by Depleting ATP Levels

**DOI:** 10.1128/AAC.02439-17

**Published:** 2018-05-25

**Authors:** Theresa O'Malley, Torey Alling, Julie V. Early, Heather A. Wescott, Anuradha Kumar, Garrett C. Moraski, Marvin J. Miller, Thierry Masquelin, Philip A. Hipskind, Tanya Parish

**Affiliations:** a TB Discovery Research, Infectious Disease Research Institute, Seattle, Washington, USA; b Department of Chemistry and Biochemistry, University of Notre Dame, Notre Dame, Indiana, USA; c Lilly Research Laboratories, Indianapolis, Indiana, USA

**Keywords:** mycobacterium, antibacterial, respiration, cytochrome oxidase, ATP, antibiotics, drug discovery, drug resistance, mycobacteria

## Abstract

The imidazopyridines are a promising new class of antitubercular agents with potent activity *in vitro* and *in vivo*. We isolated mutants of Mycobacterium tuberculosis resistant to a representative imidazopyridine; the mutants had large shifts (>20-fold) in MIC. Whole-genome sequencing revealed mutations in Rv1339, a hypothetical protein of unknown function. We isolated mutants resistant to three further compounds from the series; resistant mutants isolated from two of the compounds had single nucleotide polymorphisms in Rv1339 and resistant mutants isolated from the third compound had single nucleotide polymorphisms in QcrB, the proposed target for the series. All the strains were resistant to two compounds, regardless of the mutation, and a strain carrying the QcrB T313I mutation was resistant to all of the imidazopyridine derivatives tested, confirming cross-resistance. By monitoring pH homeostasis and ATP generation, we confirmed that compounds from the series were targeting QcrB; imidazopyridines disrupted pH homeostasis and depleted ATP, providing further evidence of an effect on the electron transport chain. A representative compound was bacteriostatic against replicating bacteria, consistent with a mode of action against QcrB. The series had a narrow inhibitory spectrum, with no activity against other bacterial species. No synergy or antagonism was seen with other antituberculosis drugs under development. In conclusion, our data support the hypothesis that the imidazopyridine series functions by reducing ATP generation via inhibition of QcrB.

## INTRODUCTION

Tuberculosis remains a serious disease of global importance, killing millions each year (1). The current treatment for disease caused by drug-sensitive bacteria is a combination regimen that includes isoniazid and rifampin, targeting bacterial cell wall synthesis and transcription, respectively (2). Newer agents, such as bedaquiline, target energy generation via inhibition of ATP synthesis. We are interested in developing new antitubercular compounds that can synergize with other agents in clinical use or under development.

A number of compound types that appear to target the electron transport chain have been identified. Among them, several related series target QcrB, a component of the terminal cytochrome oxidase (3–5). We previously investigated the imidazopyridine (IMP) series and demonstrated characteristics of the molecules that suggest they may target QcrB. The imidazopyridines have the same heterocyclic core and are structurally related to the clinical candidate Q203, which is proposed to target QcrB directly (4).

In this study, we isolated and confirmed *Mycobacterium tuberculosis* strains with mutations that confer resistance to the imidazopyridines. We also determined the effects of compound exposure on pH homeostasis and ATP depletion, which are indicative of QcrB inhibition. Finally, we demonstrate that this class of molecules has good microbiological properties, showing potent activity against extracellular and intracellular mycobacteria, as well as bacteriostatic activity against replicating bacteria.

## RESULTS

**FIG 1:**
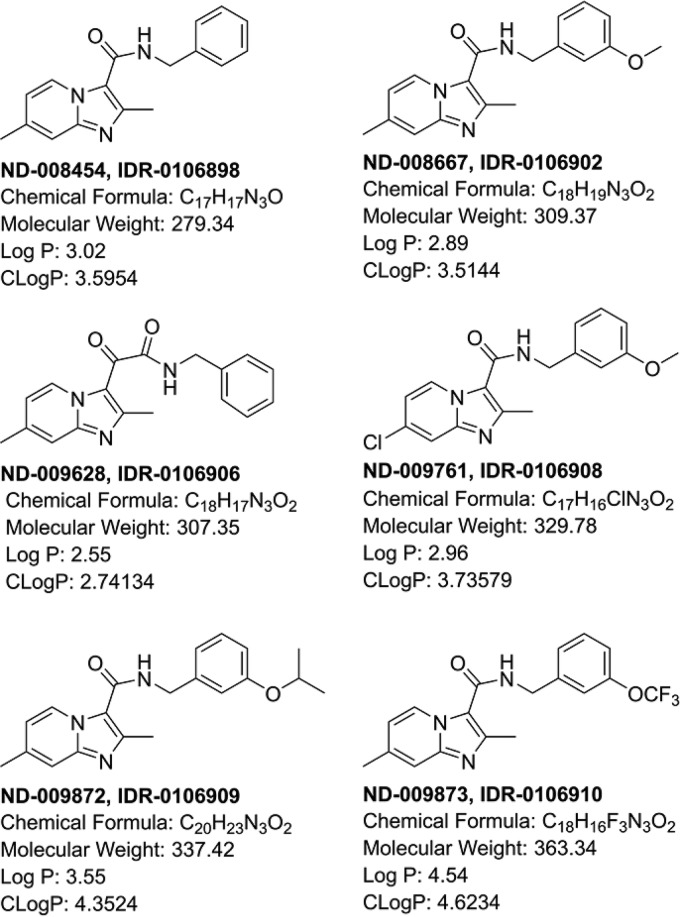
Structures of molecules used in this study.

We were interested in determining the target of the imidazopyridines (Fig. 1), a compound series that has good potency against extracellular and intracellular bacteria, as well as efficacy in a mouse model of infection against M. tuberculosis (6–13).

### Mutations in Rv1339 and QcrB confer resistance to imidazopyridines.

We isolated mutants resistant to a representative compound from the series (ND-009628) (Fig. 1). Resistance was confirmed for each mutant, and we identified three mutants with a ≥20-fold shift in the MIC (Table 1). Whole-genome sequencing of three confirmed resistant isolates revealed two different mutations in Rv1339. We confirmed mutations in Rv1339 by PCR amplification and sequencing. The mutations identified were nonsynonymous mutations of either proline to leucine or serine to proline in adjacent loci. Given the nature of these mutations, which involved changes to proline, we predicted they would result in structural changes in the protein. Since previous work suggested that the target of the imidazopyridines is QcrB, a component of the respiratory chain, we sequenced QcrB in all three resistant isolates, but there were no mutations.

**TABLE 1:** M. tuberculosis isolates with resistance to the IMP series of molecules

Compound	Strain identifier	SNP	MIC_99_ (μM)^*a*^
ND-009628	WT^*b*^	NA^*c*^	5.0
ND-009628	LP-0106906-RM1	Rv1339 P121L	>100
ND-009628	LP-0106906-RM2	Rv1339 S120P	>100
ND-009628	LP-0106906-RM3	Rv1339 P121L	100
ND-008454	WT	NA	1.25
ND-008454	LP-0106898-RM5	QcrB T313I	>10
ND-008454	LP-0106898-RM6	QcrB T313I	>10
ND-008454	LP-0106898-RM1	QcrB T313I	>10
ND-009761	WT	NA	3.1
ND-009761	LP-0106908-RM1	Rv1339 E219K	12.5
ND-009761	LP-0106908-RM3	Rv1339 S119W	12.5
ND-009761	LP-0106908-RM4	Rv1339 T14A	12.5
ND-009872	WT	NA	1.0
ND-009872	LP-0106909-RM1	Rv1339 T14A	5.0
ND-009872	LP-0106909-RM4	Rv1339 T14A	5.0
ND-009872	LP-0106909-RM5	Rv1339 T14A	5.0

a The MIC_99_s (the minimum concentrations required to prevent 99% of growth) of the indicated compounds were determined on solid medium.

b WT, wild type.

c NA, not applicable.

Since we did not expect mutations in Rv1339, we selected three additional compounds (ND-008454, ND-009761, and ND-009872) (Fig. 1) and isolated resistant mutants for characterization; resistance was confirmed on solid medium, with MIC shifts of >4-fold for each compound (Fig. 1 and Table 1). For compounds ND-009761 and ND-009872, mutations were seen in Rv1339 only. In contrast, for compound ND-008454, we found the mutation in all strains sequenced to be T313I in QcrB.

For each compound, we saw mutations either in Rv1339 or in QcrB. Therefore, we determined whether the mutations were specific to each compound or if the strains were cross-resistant to other members of the compound class (Table 2). We selected two compounds: ND-008454, for which mutations were noted in *qcrB*, and ND-009872, for which mutations were noted in Rv1339. We tested the two compounds against each of the mutant strains (one *qcrB* and five Rv1339 mutant strains). In all cases, we saw resistance with large shifts: for ND-008454, the shift was 33-fold, and for ND-009872, the shift was >8-fold. We also determined the MICs of five compounds for the QcrB_T313I_ mutant strain in liquid medium (Table 3); the strain demonstrated increased resistance to all the compounds, with at least 8-fold shifts in the MIC, and in most cases >20-fold shifts. Our data confirmed that mutations in either Rv1339 or QcrB did confer resistance to the compound class.

**TABLE 2:** M. tuberculosis mutant strains are cross-resistant to the IMP series of molecules

Strain	SNP	MIC_99_ (μM)^*a*^	
		ND-008454	ND-009872
WT	NA	0.30	1.3
LP-0106898-RM5	QcrB T313I	10	>10
LP-0106906-RM1	Rv1339 P121L	10	>10
LP-0106906-RM2	Rv1339 S120P	10	>10
LP-0106908-RM1	Rv1339 E219K	10	>10
LP-0106908-RM3	Rv1339 S119W	10	>10
LP-0106909-RM1	Rv1339 T14A	10	>10

a The MIC_99_s of the indicated compounds were determined on solid medium.

**TABLE 3:** The M. tuberculosis QcrBT_313I_ mutant strain is resistant to the IMP series of molecules

Compound	MIC_90_ (μM)^*a*^	
	WT	QcrB_T313I_
ND-009873	0.32	7.5
ND-009872	0.37	6.5
ND-009761	2.4	>20
ND-008667	0.56	12
ND-008454	0.46	>20

a The MIC_90_s of the indicated compounds were determined on liquid medium.

### Imidazopyridines disrupt pH homeostasis in M. tuberculosis.

**FIG 2:**
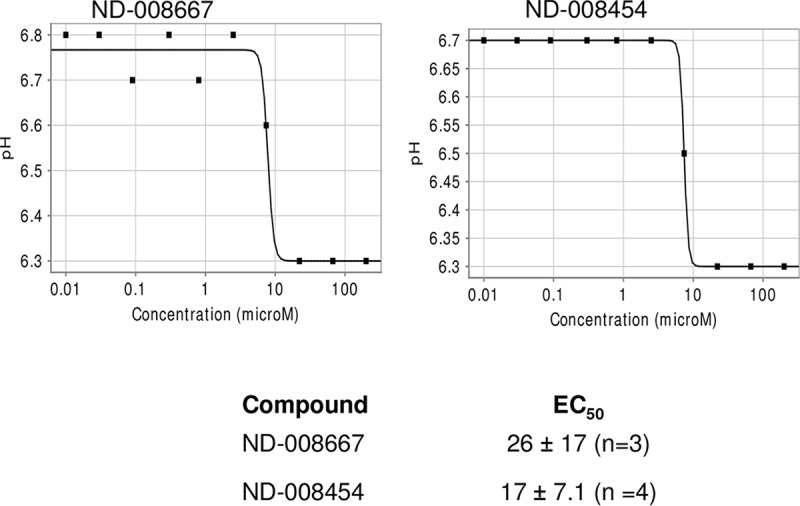
IMPs disrupt pH homeostasis in M. tuberculosis. The internal pH was measured in M. tuberculosis maintained in phosphocitrate buffer at pH 4.5 after 2 days of exposure to compounds using a ratiometric GFP. Representative curves are shown for two compounds. The 50% effective concentration (EC_50_) (the concentration at which a half-maximal effect was seen) was measured for each compound; the values for multiple replicate experiments (*n*) were reported as means ± standard deviations.

Our data supported the idea that the IMP series targets QcrB, a key component of the electron transport chain. However, since we identified single nucleotide polymorphisms (SNPs) in Rv1339 that also conferred resistance, we looked at the effects of compounds on cellular processes controlled by respiration. QcrB is a proton-pumping member of the electron transport chain, and its inhibition would be predicted to disrupt the pH gradient across the membrane. Therefore, we looked at the ability of compounds to disrupt pH homeostasis. We used a ratiometric green fluorescent protein (GFP) to monitor the intracellular pH (pH_IB_) (14) in M. tuberculosis exposed to compounds over 2 days in acidic medium. We defined activity as the ability to reduce the pH_IB_ below 6.5. Both compounds were able to disrupt pH homeostasis in a dose-dependent fashion (Fig. 2).

### Imidazopyridines deplete intracellular ATP levels in M. tuberculosis.

**FIG 3:**
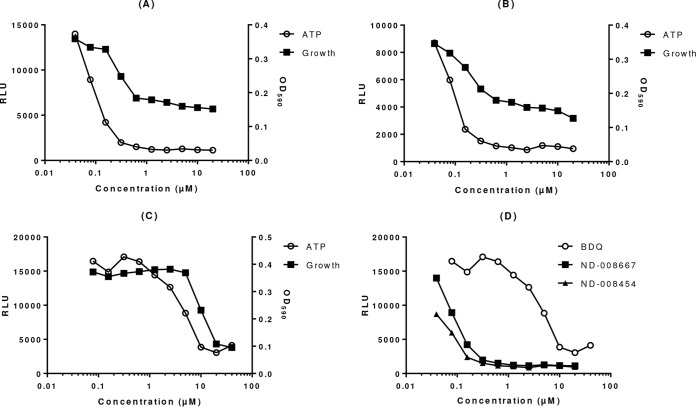
IMPs lead to ATP depletion in M. tuberculosis. ATP levels were measured in M. tuberculosis exposed to compounds for 24 h. The optical densities at 590 nm (OD_590_) of the cultures were measured to determine growth. (A) ND-008667. (B) ND-008454. (C) Bedaquiline. (D) ATP depletion for all three compounds.

In addition to effects on the maintenance of the proton gradient, we expected that inhibition of QcrB would have a direct effect on ATP levels. We predicted that, similar to the ATP synthase inhibitor bedaquiline, ATP levels would be depleted in a dose-dependent manner after exposure to IMP compounds. We tested this and demonstrated that, indeed, we did see depletion of ATP (Fig. 3).

### Imidazopyridines are bacteriostatic against replicating M. tuberculosis.

**FIG 4:**
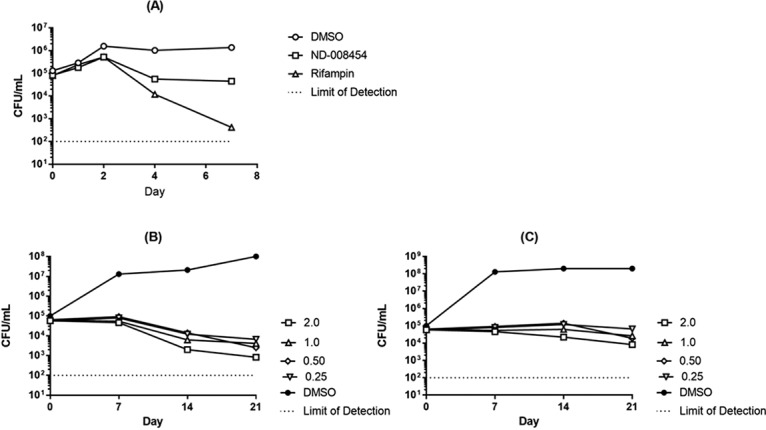
A representative IMP has time-dependent killing activity against M. tuberculosis. (A) M. tuberculosis was exposed to 10× MIC over 7 days (2 μM ND-008454 or 30 nM rifampin). (B and C) M. tuberculosis was exposed to increasing concentrations of ND-008454 over 21 days in aerobic culture. Bacterial viability was measured as CFU by serial dilution and plating onto solid medium; colonies were counted after 3 to 4 weeks.

We are interested in the potential of imidazopyridines as novel antitubercular agents. We previously demonstrated potent inhibitory activity of compounds from this series against M. tuberculosis in liquid culture (8, 13). We determined whether this inhibitory activity could translate into loss of bacterial viability. We monitored bacterial viability by CFU under aerobic, replicating growth conditions using a representative compound (ND-008454). We first compared activity levels over 7 days at 10× MIC; the compound inhibited growth but showed little killing in this short time (Fig. 4A). In contrast, the frontline drug rifampin showed demonstrable killing, with >2-log-unit decreases in viable bacteria over 7 days. We repeated the experiment over a longer time course with different compound exposures (Fig. 4B and C). Over 21 days, we saw a minimal kill rate of 1 to 2 log units at 10× MIC. The kill rate was independent of the concentration; thus, the kill kinetics are time dependent. According to standard guidelines from the Clinical and Laboratory Standards Institute (15), the compounds are bacteriostatic against replicating bacteria.

### Imidazopyridines have potent activity against intracellular M. tuberculosis.

The ability of compounds to penetrate into eukaryotic cells and act against intracellular M. tuberculosis is a desirable characteristic in novel drugs. We tested the intracellular activities of the compounds in this study (Table 4). All the compounds were active, with good potency (IC_90_s ranged from 1.8 to 13 μM). In parallel, we tested for cytotoxicity against the macrophage line; none of the compounds were cytotoxic against the THP1 cells (50% toxic concentration [TC_50_], >50 μM). These are promising properties for new antitubercular agents. We also tested for cytotoxicity against HepG2 cells in glucose medium; all the compounds were inactive, with TC_50_s of >100 μM.

**TABLE 4:** IMP series activity against intracellular M. tuberculosis

Compound	IC_50_ (μM)	IC_90_ (μM)	TC_50_^*a*^ (μM)
ND-008667	0.15	3.1	>50
ND-009628	1.7	8.5	>50
ND-009872	0.39	1.8	>50
ND-009761	3.0	13	>50
ND-008454	0.20	3.0	>50

a The TC_50_s (50% toxic concentrations; concentrations of compounds required to inhibit growth by 50%) were determined for uninfected THP-1 cells.

### Imidazopyridines are inactive against other species.

We determined the spectrum of activity for the series. None of the compounds we tested were active against the closely related organism Mycobacterium smegmatis (Table 5). We tested two compounds against representative Gram-negative (Escherichia coli) and Gram-positive (Staphylococcus aureus) species, but none had any activity (Table 5). This suggests that the series has a narrow spectrum and is largely selective for M. tuberculosis.

**TABLE 5:** IMP series activity against other bacteria

Compound	MIC_99_ (μM)^*a*^			
	M. smegmatis	E. coli	S. aureus	M. tuberculosis
ND-008454	>200	>10	>10	1.25
ND-009872	>200	>100	>100	0.50
ND-008667	>200	NT	NT	1.3
ND-009628	>200	NT	NT	5.0

a The MIC_99_s of the indicated compounds were determined on solid medium. NT, not tested.

### Imidazopyridines do not have synergy with other agents.

We tested whether the IMP series had any synergy with other agents under development; we tested ND-008454 with PA824 (16), BTZ043 (17), SQ109 (18), and amoxicillin, representing inhibitors with different targets. The compounds were tested in a classic checkerboard assay to determine the fractional inhibitory concentration index (FICI). For each combination tested, we saw neither synergy nor antagonism, with FICIs of 2 (data not shown).

## DISCUSSION

Previous work has proposed that QcrB is the target of molecules with structural similarity to the imidazopyridines, and our work has demonstrated compound profiles consistent with this hypothesis. Surprisingly, we identified a number of mutations in Rv1339 that have not previously been associated with resistance to structural homologs, such as Q203. The SNPs observed suggest that they would result in structural changes to the protein. Rv1339 is a member of the β-lactamase superfamily, so the protein structure does not give much insight into its possible role in the cell, but it could play a role in cell wall synthesis, and mutations could lead to structural alterations that affect compound penetration. In support of this, the protein is located in the membrane fraction (19), and the potency of Q203 is affected by efflux (20).

We have previously isolated strains with different mutations in Rv1339 (V90M or Y94C) that are resistant to another series of molecules that also target QcrB, the phenoxyalkylbenzimidazoles. In the latter case, the level of resistance conferred was low in comparison to the level of resistance seen in strains with QcrB mutations, and resistance to all molecules in the class was not conferred. Thus, we discounted the possibility that Rv1339 was the target of the phenoxyalkylbenzimidazoles. However, in this study, we saw high-level resistance to all the compounds conferred by mutations in Rv1339, as well as in QcrB. Since the IMP compounds disrupt pH homeostasis and ATP generation, we propose that their target is QcrB and that inhibition of QcrB is the mode of action. We think it unlikely that Rv1339 is the primary target of the IMP series but hypothesize that it could have an effect on cell wall or cell envelope structure, thereby impacting compound permeation or export.

A representative compound from the series showed bacteriostatic activity against replicating bacteria in aerobic culture. This is consistent with the profile we have seen for other QcrB inhibitors. It is of interest, that despite this lack of rapid killing activity *in vitro*, IMPs and related compounds, such as Q203, are effective against *M. tuberculosis in vivo*. Although we did not see synergy with IMPs and other agents in growth inhibition assays, we have recently demonstrated that QcrB inhibitors can synergize with other agents when bacterial kill rates are measured and that this potentiation can be seen against replicating bacteria. It would be interesting to determine if the IMP series has similar properties and so could be developed as a key component of a new drug regimen.

## MATERIALS AND METHODS

### Compounds.

The syntheses of all the imidazopyridines described in this study have been previously reported (6–13). BTZ043, PA824, SQ109, and bedaquiline were synthesized in house for use.

### Determination of MICs.

MICs for M. tuberculosis cultured aerobically in Middlebrook 7H9 medium supplemented with 10% (vol/vol) oleic acid–bovine serum albumin (BSA)–d-glucose–catalase (OADC) (Becton Dickinson) and 0.05% (wt/vol) Tween 80 (7H9-Tw-OADC) were determined in liquid medium after 5 days of growth, as described previously (13). For synergy testing, compound combinations were tested in checkerboard fashion in 96-well plates; the compounds were diluted serially either in the *x* or *y* axis. Growth inhibition curves were fitted using the Levenberg-Marquardt algorithm, and MICs were reported as the concentrations required to inhibit growth by 90% (IC_90_s). For synergy, the FICI was defined as follows: FICI = FIC_A_ + FIC_B_, where FIC_A_ is the MIC of compound A in the presence of compound B divided by the MIC of compound A alone and FIC_B_ is the MIC of compound B in the presence of compound A divided by the MIC of compound B alone. An FICI of ≤0.5 is synergistic, and an FICI of ≥4 is antagonistic (21). MICs in solid medium were determined by plating on Middlebrook 7H10 medium plus 10% (vol/vol) OADC-containing compound and incubating them for 3 to 4 weeks before scoring growth in 96-well plates (MIC) or counting CFU on 90-mm petri plates (MIC_99_).

### Isolation and characterization of resistant mutants.

Spontaneous resistant mutants were isolated as described previously, and resistance was confirmed on solid medium (22). Briefly, 10^7^ to 10^9^ bacteria were plated at 5× to 10× MIC on solid medium. Colonies appearing after 3 to 6 weeks were cultured and tested for resistance. Whole-genome sequencing was carried out on confirmed resistant isolates (EdgeBio). Mutations were confirmed by sequencing of PCR-amplified products from chromosomal DNA.

### Determination of eukaryotic cytotoxicity.

Cytotoxicity was tested against the HepG2 cell line (ATCC) as described previously (23). Compounds were tested as 10-point, 3-fold serial dilutions in dimethyl sulfoxide (DMSO) (final assay concentration, 1% DMSO), and viability was measured using CellTiter-Glo (Promega). Inhibition curves were fitted using the Levenberg-Marquardt algorithm. The TC_50_ was defined as the concentration required to reduce cell viability by 50% after 2 days of incubation.

### Determination of intrabacterial pH.

M. tuberculosis expressing ratiometric GFP (14) was resuspended in phosphate citrate buffer, pH 4.5, containing 0.05% (wt/vol) tyloxapol and exposed to serial dilutions of compounds in DMSO (final concentration, 2% DMSO) for 2 days. Fluorescence was measured at excitation/emission wavelengths of 400/516 and 485/516 nm (14). The ratio was calculated, and the intrabacterial pH was calculated from the standard curve.

### Measurement of intrabacterial ATP levels.

M. tuberculosis was exposed to compounds for 24 h. ATP levels were measured using the BacTiter-Glo assay kit (Promega) and expressed as relative luminescence units (RLU).

### Kill kinetics.

For aerobic, replicating conditions, late-log-phase bacteria were inoculated into 7H9-Tw-OADC medium containing compounds (final DMSO concentration, 2%) at 10^5^ to 10^6^ CFU/ml. CFU were determined by plating 10-fold serial dilutions onto Middlebrook 7H10 agar containing 10% (vol/vol) OADC (7H10-OADC), and colonies were counted after 3 to 4 weeks.

### Intracellular activity.

THP-1 cells were cultured in complete RPMI (RPMI 1640, 10% fetal bovine serum [FBS], 2 mM Corning Glutagro, 1 mM sodium pyruvate) and differentiated into macrophage-like cells using 80 nM phorbol 12-myristate 13-acetate (PMA) overnight at 37°C, 5% CO_2_. THP-1 cells were infected with M. tuberculosis expressing luciferase (24) at a multiplicity of infection (MOI) of 1 in complete RPMI overnight. Infected cells were washed and seeded at 4 × 10^4^ cells per well in 96-well plates. The compounds were tested as a 10-point, 3-fold dilution series (final DMSO concentration, 0.5%). Infected cells were exposed to the compounds for 3 days in a humidified atmosphere of 37°C and 5% CO_2_. Bacterial growth was measured in RLU. Growth inhibition curves were fitted using the Levenberg-Marquardt algorithm; the IC_50_ and IC_90_ were defined as the concentrations resulting in 50% and 90% inhibition of intracellular growth, respectively.

### THP1 cytotoxicity.

Uninfected THP1 cells were cultured in complete RPMI and differentiated into macrophage-like cells using 80 nM PMA overnight at 37°C and 5% CO_2_. The cells were seeded and exposed to the compounds as described above. Macrophage viability was measured in RLU using CellTiter-Glo (Promega). The TC_50_ was defined as the compound concentration that resulted in 50% viability.

### Spectrum.

MICs were determined using the serial dilution agar method. S. aureus RN4220 and E. coli DH5α were grown on LB agar and M. smegmatis mc^2^155 on Middlebrook 7H10 agar plates plus 10% (vol/vol) OADC supplement. A late-log-phase culture was plated (10^5^ CFU/ml) and incubated at 37°C until large colonies formed on the no-compound control plates. The MIC_99_ was defined as the lowest concentration of compound that had less than 1% growth.
